# Supplementary material for: The Effect of Non-Overlapping Somatic Mutations in BRAF, NRAS, NF1, or CKIT on the Incidence and Outcome of Brain Metastases during Immune Checkpoint Inhibitor Therapy of Metastatic Melanoma
Source: Cancers (Basel). 2024 Jan 30;16(3):594. doi: 10.3390/cancers16030594 (PMC10854687; doi:10.3390/cancers16030594)
Supplement: Supplementary file 1 [file cancers-16-00594-s001.zip › NH Supplemental Table 1 10-2-23.pdf]

Supplemental table S1: Patient demographics

UPN	Mut	Age	Sex	Race	Primary site	Site of metastases	Stage	Initial LDH	TMB (per Mb)	PDL1	Brain mets
1	T599_V600insT	55	M	C	extr	LN, lung	IVB	172			yes-d
2	BRAF-NF2 fusion	53	F	C	UNK	LN, adrenal	IVC	115	6	6	yes-d
3	BRAF deletion	66	M	C	extr	bone	IVC	162	5	5	N
4	CCDC127-BRAF fusion	64	M	C	trunk	LN, liver, spleen, bone	IVC	190			N
5	exon 8 rearrang	66	F	C	scalp	abd, bone	IVC	187	5	5	N
6	BRAF V600E	53	M	C	trunk	LN, SQ, brain	IVD	286	8	8	yes-o
7	BRAF V600E	45	M	C	UNK	brain	IVD	799	13	13	yes-o
8	BRAF V600E	59	F	C	extr	brain, LN, abd	IVD	394	6	6	yes-o
9	BRAF V600E	70	F	C	UNK	brain	IVD	203	5	5	yes-o
10	BRAF V600K	52	F	C	trunk	LN, brain	IVD	164	19	19	yes-o
11	BRAF V600K	72	F	C	trunk	LN, brain	IVD	226	11	11	yes-o
12	BRAF V600R	62	F	C	UNK	LN, brain	IVD	276	11	11	yes-o
13	BRAF V600E	34	M	C	neck	SQ, liver, spleen lung, brain	IVD	251	24	24	yes-o
14	BRAF V600E	43	F	C	trunk	adrenal, lung	IVC	223	6	6	yes-d
15	BRAF V600E	26	M	C	trunk	LN, adrenal, bone	IVC	216	5	5	N
16	BRAF V600E	50	F	C	trunk	LN (unresectable)	IIIB	161	10	10	N
17	BRAF V600E	59	M	C	trunk	lung, bone	IVC	254	3	3	N
18	BRAF V600E	48	F	C	trunk	LN	IVA	163	3	3	N
19	BRAF V600E	51	F	C	extr	LN, lung	IVB	220	indet	indet	N
20	BRAF V600E	59	M	C	trunk	LN	IVA	167			N
21	BRAF V600E	48	M	C	extr	SQ	IVA	184	8	8	N
22	BRAF V600E	56	M	C	trunk	SQ, abd, bone	IVC	221	9	9	N
23	BRAF V600E	87	M	C	trunk	SQ, liver, LN, bone	IVC	359	14	14	N
24	BRAF V600E	66	F	C	extr	LN, SQ	IVA	196	8	8	N
25	BRAF V600E	36	M	C	scalp	LN,SQ	IVA	171	1	1	N
26	BRAF V600E	63	F	C	trunk	mucosal, lung	IVC	172	11	11	N
27	BRAF V600E	35	F	C	trunk	SQ	IVA	160	14	14	N
28	BRAF V600E	79	F	C	extr	SQ, liver	IVC	189	5	5	N
29	BRAF V600E	52	F	C	trunk	LN	IVA	109	78	78	N
30	BRAF V600E	68	F	C	face	SQ, lung	IVB	184			N
31	BRAF V600K	75	M	C	face	SQ	IVA	133	44	44	N
32	BRAF V600K	68	M	C	trunk	SQ, lung	IVB	201	50	50	N
33	BRAF V600K	55	M	C	ear	SQ, LN	IVA	189	32	32	N
34	CKIT A829P	72	M	C	trunk	SQ, lung	IVB	163	>10	>10	yes-d
35	NF1	77	M	C	scalp	lung, liver, bone, brain	IVD	463	82	82	yes-o
36	NF1	67	M	C	trunk	liver, brain	IVD	312	79	79	yes-o
37	NF1	69	M	C	UNK	brain, lung SQ	IVD	236	n/a	n/a	yes-o
38	NF1	59	M	C	trunk	lung, bone	IVC	120	n/a	n/a	yes-d
39	NF1	48	M	C	face	lung	IVB	196	139	139	N
40	NF1	83	M	C	trunk	SQ, lung	IVB	228			N
41	NF1	84	M	C	trunk	lung, peritoneal, bone, renal, adrenal, pleura	IVC	1024	61	61	N
42	NF1	100	F	C	neck	LN,SQ	IVA	217	34	34	N
43	NF1	79	M	C	UNK	pancreas	IVC	281	106	106	N
44	NF1	75	M	C	scalp	liver, brain	IVC	153	47	47	N
45	NF1	99	M	C	scalp	SQ, lung. bone	IVC	154	6	6	N
46	NF1	75	F	C	UNK	lung	IVB	364	155	155	N
47	NF1	68	F	C	trunk	LN, SQ	IVA	297	54	54	N
48	NF1	71	M	C	face	LN, SQ	IVA	145	34	34	N
49	NF1	71	M	C	scalp	LN	IVA	154	73	73	N
50	NF1	48	M	C	face	SQ, LN	IVA	214	5	5	N

51	NF1	53	F	C	extr	SQ, LN	IVA	268	0	0	N
52	NF1	86	M	C	scalp	lung	IVB	176	78	78	N
53	NF1	79	M	C	scalp	SQ	IVA	215	154	154	N
54	NF1	75	M	C	trunk	bone	IVC	213			N
55	NF1	81	M	C	trunk	LN, SQ, lung	IVB	166	52	52	N
56	NF1	77	M	C	face	SQ	IVA	269	82	82	N
57	NF1	64	M	C	extr	SQ, lung	IVB	216	49	49	N
58	NRAS Q61R	63	F	C	trunk	LN, liver, lung	IVC	298	11	11	yes-d
59	NRAS Q61R	51	M	C	trunk	SQ	IVA	127	8	8	N
60	NRAS Q61K	49	M	C	extr	SQ	IVA	191	57	57	N
61	NRAS Q61K	32	M	C	UNK	LN, lung	IVB	239	13	13	N
62	NRAS Q61K	79	M	C	UNK	LN	IVA	499	18	18	N
63	NRAS Q61K	69	M	C	trunk	abd	IVC	180	4	4	N
64	NRAS Q61K	66	M	C	trunk	LN, lung	IVB	154	25	25	N
65	NRAS Q61L	57	M	C	UNK	SQ, muscle	IVA	171	32	32	N
66	NRAS Q61L	68	M	C	extr	SQ	IVA	133	39	39	N
67	NRAS Q61R	48	F	C	extr	SQ, renal, lung	IVC	131	n/a	n/a	N
68	NRAS Q61R	65	M	C	trunk	LN	IVA	184	15	15	N
69	NRAS Q61R	67	M	C	trunk	LN, pleura	IVB	205	21	21	N
70	NRAS Q61R	71	M	C	UNK	LN, spleen, liver	IVC	141	indet	indet	N
71	NRAS Q61R	41	M	C	extr	LN, SQ	IVA	167			N
72	NRAS G12D, T50I	72	M	C	trunk	LN	IVA	171	81	81	N
73	QN	70	M	C	scalp	lung	IVB	305	88	88	N
74	QN	56	M	H	extr	liver, bone	IVC	187	2	2	N
75	QN	63	M	C	scalp	lung, LN	IVB	191	16	16	N
76	QN	63	M	C	trunk	LN, ascites	IVC	160	4	4	N
77	QN	75	F	C	extr	LN, SQ	IVA	202	24	24	N
78	QN	57	M	C	extr	LN, bone	IVC	286	11	11	N
79	QN	60	M	C	trunk	lung	IVB	193	indet	indet	N
80	QN	42	M	C	extr	LN,SQ	IVA	NA	1	1	N
81	QN	51	F	C	trunk	LN	IVA	293	9	9	N
82	QN	76	F	A	extr	LN	IVA	181	1	1	N
83	QN	80	M	C	extr	lung, LN	IVB	142			N
84	QN	76	F	C	neck	lung	IVB	181			N
85	QN	44	M	C	ear	LN, liver, lung	IVC	240	19	19	N

UPN, unique patient number; QN, “quadruple negative” (no BRAF, NRAS, CKIT or NF1 mutations identified); F, female; M, male; C, Caucasian; H, Hispanic; A, Asian; extr, extremity; UNK, unknown primary; SQ, subcutaneous; LN, lymph node; abd, abdomen; yes-d; delayed onset of brain metastases; yes-o, brain metastases at diagnosis of metastatic disease; N, no brain metastases.
