# Supplementary material for: The Effect of Non-Overlapping Somatic Mutations in BRAF, NRAS, NF1, or CKIT on the Incidence and Outcome of Brain Metastases during Immune Checkpoint Inhibitor Therapy of Metastatic Melanoma
Source: Cancers (Basel). 2024 Jan 30;16(3):594. doi: 10.3390/cancers16030594 (PMC10854687; doi:10.3390/cancers16030594)
Supplement: Supplementary file 1 [file cancers-16-00594-s001.zip › NH Supplemental Table 2 .pdf]

Supplemental table S2: Treatment outcomes

UPN	Mutation	Initial ICI regimen	ICI doses	PFS (mo)	TT added	Brain mets	CNS RT	OS (mo)	CKI toxicity	Current status
1	T599_V600insT	nivo	7	4.3	DT, EB	yes-d	NT	14.1	immune nephritis	DOD
2	BRAF-NF2 fusion	ipi	25	6.0	DT	yes-d	NT	57.3	hypothyroidism	DOD
3	BRAF deletion	pembro	8	19.0		N		22.1	hypothyroidism	DOD
4	CCDC127-BRAF fusion	nivo	12	4.1		N		8.5	diarrhea	DOD
5	exon 8 rearrang	ipi+nivo	8	30.2	DT	N		50.8		DOD
6	BRAF V600E	ipi+nivo	9	2.5	EB	yes-o	WBRT	9.8		DOD
7	BRAF V600E	ipi	8	21.1	EB	yes-o	NT	116.0	diarrhea	NED
8	BRAF V600E	ipi+nivo	6	9.1		yes-o	SRS	10.0	rash, colitis, encephalopathy	NED
9	BRAF V600E	ipi+nivo	11	10.9		yes-o	SRS	10.9	rash,	NED
10	BRAF V600K	ipi+nivo	10	9.6		yes-o	SRS	22.3	hypothyroidism	NED
11	BRAF V600K	ipi+nivo	6	7.9		yes-o	NT	9.7	rash, colitis, ankle swelling, fatigue, hypopituitarism	NED
12	BRAF V600R	nivo	7	7.1		yes-o	WBRT	7.8	pneumonitis	DOD
13	BRAF V600E	nivo	12	27.6	EB	yes-o	WBRT	35.5		DOD
14	BRAF V600E	ipi+nivo	7	6.7	EB	yes-d	SRS	17.4	fevers	DOD
15	BRAF V600E	ipi+nivo	13	29.7	B	N		47.1	Fever, diarrhea	NED
16	BRAF V600E	nivo	8	1.8	DT, EB	N		43.5	skin rash	NED
17	BRAF V600E	ipi+nivo	16	27.8		N		39.9		DOD
18	BRAF V600E	nivo	12	25.4	DT	N		37.1	hypothyroidism	NED
19	BRAF V600E	nivo	9	42.2		N		42.5	rash, hypothyroidism	NED
20	BRAF V600E	ipi	6	8.5	Vem, D	N		24.2	colitis	DOD
21	BRAF V600E	ipi+nivo	4	16.2		N		26.7	hypopituitarism, uveitis	NED
22	BRAF V600E	ipi+nivo	8	11.1		N		15.6	CIDP	NED
23	BRAF V600E	ipi+nivo	3	2.0		N		2.3		DOD
24	BRAF V600E	ipi+nivo	8	10.0		N		22.3	rash, hepatitis, hypothyroid, dry eyes and mouth	NED
25	BRAF V600E	ipi+nivo	5	1.5	EB	N		21.6		NED
26	BRAF V600E	ipi+nivo	4	9.3		N		21.4	encephalopathy, hypothyroid, rash	DOD
27	BRAF V600E	ipi+nivo	2	9.8		N		22.5	colitis, pneumonitis	Ongoing
28	BRAF V600E	nivo	3			N		3.0	AKI	Died other
29	BRAF V600E	ipi+nivo	13	11.2		N		12.1	abdominal cramps, N/V	NED
30	BRAF V600E	ipi+nivo	3	2.3		N		10.1		NED
31	BRAF V600K	nivo	12	24.9		N		36.5		NED
32	BRAF V600K	ipi+nivo	7	33.6		N		41.5	hypopituitarism, rash	NED
33	BRAF V600K	ipi+nivo	7	34.3		N		41.3	hypopituitarism	NED
34	CKIT A829P	ipi+nivo	17	7.3	nilot	yes-d	WBRT	6.2	hot flash, fatigue, colitis, fever, rash, hypothyroidism	DOD
35	NF1	ipi+nivo	4	2.0		yes-o	NT	2.5	diarrhea, rash	DOD
36	NF1	ipi+nivo	3	1.8	T	yes-o	SRS	2.7	hypothyroid	DOD
37	NF1	pembro	5	5.7		yes-o	WBRT	7.9	uveitis	DOD
38	NF1	ipi+nivo	4	3.2		yes-d	NT	5.3	colitis	DOD
39	NF1	ipi+nivo	11	35.5		N		46.7	fevers	NED
40	NF1	nivo	7	12.4		N		12.4		NED
41	NF1	ipi+nivo	1	1.0		N		1.2		DOD
42	NF1	nivo	3	39.5		N		47.7		Died other
43	NF1	ipi+nivo	10	14.6		N		26.6		NED
44	NF1	nivo	26	47.4	Su	N		59.7		NED
45	NF1	nivo	12	2.3	T, B	N		11.1		DOD
46	NF1	pembro	9	42.4		N	SRS	51.5	hypothyroidism	NED
47	NF1	nivo	16	3.9	T	N		45.2		NED
48	NF1	nivo	10	8.3		N		9.3		Died other
49	NF1	nivo	12	40.7		N		52.8	colitis	NED
50	NF1	ipi+nivo	9	13.4	T	N		45.3	cutaneous granulomas; flank pain, dizziness	NED
51	NF1	nivo	7	3.0		N		23.1	hypothyroidism	DOD

52	NF1	nivo	9	9.2	T	N		23.0		NED
53	NF1	ipi+nivo	4	8.7		N		17.2	rash	NED
54	NF1	ipi+nivo	6	41.3		N		41.6	colitis, back pain,	DOD
55	NF1	pembro	13	34.6		N		23.0	hypothyroidism	NED
56	NF1	nivo	15	17.9	T	N		58.1		NED
57	NF1	nivo	3	1.9		N		57.4	colitis, peripheral neuropathy	NED
58	NRAS Q61R	ipi+nivo	7	2.9		yes-d	NT	8.5	rash, hypophysitis, diarrhea	DOD
59	NRAS Q61R	ipi+nivo	16	14.5	T, B	N		27.0	colitis, arthritis	DOD
60	NRAS Q61K	ipi+nivo	10	19.6		N		35.2		NED
61	NRAS Q61K	ipi+nivo	11	4.7	T	N		29.5	colitis	DOD
62	NRAS Q61K	nivo	7	13.0		N		24.5		NED
63	NRAS Q61K	ipi+nivo	7	4.2	B	N		17.6		DOD
64	NRAS Q61K	ipi+nivo	9	11.3		N		13.6	colitis, sinus infection, renal stones, anemia,	Ongoing
65	NRAS Q61L	ipi+nivo	11	38.2		N		45.4	colitis, hyperthyroidism	NED
66	NRAS Q61L	nivo	9	21.0		N		23.0	fatigue, arthralgia	NED
67	NRAS Q61R	pembro	5	11.4	T	N		27.4	diarrhea, rash, hypopituitarism	DOD
68	NRAS Q61R	ipi+nivo	2	24.9		N		25.5	rash, colitis, worsening RA	NED
69	NRAS Q61R	pembro	30	9.9	T	N		23.6	colitis	DOD
70	NRAS Q61R	nivo	10	9.9		N		21.5		NED
71	NRAS Q61R	ipi+nivo	7	8.6		N		8.6		NED
72	NRAS G12D, T50I	nivo	3	32.4	T	N		44.4		NED
73	QN	ipi+nivo	10	10.2		N		21.8	rash, hypothyroidism	NED
74	QN	ipi+nivo	12	39.9	T	N		39.9		DOD
75	QN	nivo	5	7.4		N		11.8	colitis	DOD
76	QN	ipi+nivo	11	3.0		N		32.3		NED
77	QN	ipi+nivo	6	23.6		N		24.0	rash, arthritis, psoriasis, pruritis, ILD	Died other
78	QN	ipi+nivo	8	11.0	T, B	N		11.2	hypothyroidism, rash, pruritis, fever, SOB, diarrhea	DOD
79	QN	ipi+nivo	4	1.9		N		2.3	hematuria	NED
80	QN	pembro	42	52.2		N		63.9		NED
81	QN	ipi+nivo	7	19.2		N		19.2	rash, colitis, hepatitis, myopathy, neuropathy, nausea	DOD
82	QN	ipi+nivo	3	6.3		N		6.4	elevated liver enzymes, rash, psoriasis	DOD
83	QN	nivo	6	7.6		N		7.8	rash, bullous pemphigoid, dizziness, nephritis, SOB	DOD
84	QN	ipi+nivo	16	13.3		N		14.6	headache, inflammatory colitis,	Died other
85	QN	ipi+nivo	7	7.8		N		7.8		NED

UPN, unique patient number; QN, “quadruple negative (no BRAF, NRAS, NF1 or CKIT mutations); nivo, nivolumab; ipi, ipilimumab; pembro, pembrolizumab; ipi+nivo, combined ipilimumab plus nivolumab; D, dabrafenib; T, trametinib, E, encorafenib; B, binimetinib; vem, vemurafeniv; nilot, nilotinib; Su, sunitnib; yes-d; delayed onset of brain metastases; yes-o, brain metastases at diagnosis of metastatic disease; N, no brain metastases; NT, not treated; WBRT, whole brain radiotherapy; SRS, stereotactic radiosurgery; CIDP, chronic immune demyelinating polyneuropathy; AKI, acute kidney injury; N/V, nausea and vomiting; RA, rheumatoid arthritis; ILD, interstitial lung disease; SOB, short of breath, DOD, died of disease; NED, no evidence of disease; Died other, died of non-cancer related causes.
